# Association of Pharmacogenotyping and Patient-Reported Outcomes in Chronic Pain Management

**DOI:** 10.1177/11786329251356560

**Published:** 2025-07-12

**Authors:** Anna Bollinger, Kurt E. Hersberger, Julia Gianora, Isabelle O. Urdieux, Henriette E. Meyer zu Schwabedissen, Nikki Rommers, Matthias Schwenkglenks, Céline K. Stäuble, Samuel S. Allemann

**Affiliations:** 1 Pharmaceutical Care Research Group, Department of Pharmaceutical Sciences, University of Basel, Basel, Switzerland; 2 Biopharmacy, Department of Pharmaceutical Sciences, University of Basel, Basel, Switzerland; 3 Department of Clinical Research, University of Basel, University Hospital Basel, Basel, Switzerland; 4 Institute of Pharmaceutical Medicine (ECPM), University of Basel, Basel, Switzerland; 5 Health Economics Facility, Department of Public Health, University of Basel, Basel, Switzerland; 6 Institute of Hospital Pharmacy, Stadtspital Zurich, Zurich, Switzerland

**Keywords:** PGx-guided therapy, HRQoL, pain intensity, pre-post-analysis, MID, clinical utility

## Abstract

**Background:**

Chronic pain is a complex condition affecting patients’ health-related quality of life (HRQoL). Pharmacogenetic (PGx) testing offers an approach to personalize pain management by optimizing medication regimens. However, the impact of this approach on measurable patient reported outcomes (PROs) remains unexplored.

**Objectives:**

This study evaluated the association of PGx testing on PROs in chronic pain patients and investigated differences between those who received PGx-guided therapy and those who did not, focusing on changes in HRQoL and pain intensity from pre-to-post PGx.

**Design:**

An exploratory pre-post analysis was conducted as part of an observational case series assessing the influence of PGx testing and subsequent PGx-guided therapy on PROs in chronic pain patients with drug-related problems under their analgesic regimen.

**Methods:**

PROs were assessed in 29 patients pre-PGx (baseline) and post-PGx (follow-up, 4-6 weeks later). HRQoL was measured using the EQ-5D-5L. The EQ index was calculated using the German value set. Pain intensity was determined with the Numeric Rating Scale (NRS). Minimal important difference (MID) threshold was applied for both outcomes. Statistical analyses included Wilcoxon signed-rank tests, chi-square tests, and effect size calculations.

**Results:**

The mean EQ index score improved from pre-to-post PGx (0.379 ± 0.420-0.697 ± 0.307, *P* < .001, *d* = −0.84). Stratification revealed that the PGx-guided therapy group showed significantly greater improvements in HRQoL and NRS compared to the non-PGx guided therapy group (*P* < .01). Among 19 patients who met the MID for the EQ index, 18 had undergone PGx-guided therapy. For NRS, MID was reached in 3 pain intensity categories in the PGx-guided therapy group.

**Conclusions:**

HRQoL and pain intensity significantly improved after PGx testing, with potentially clinically relevant results in the PGx-guided therapy group. Due to the observational nature of the study, further controlled studies are required to assess the clinical impact and economic feasibility of PGx-guided therapy.

## Introduction

Chronic pain is a pervasive condition and represents a significant global public health burden, affecting more than 1 billion individuals worldwide by impacting their quality of life, daily functioning, mental, and social well-being.^1,2^ The heterogeneity of chronic pain conditions as well as the variability in drug responses, pose a considerable challenge to effective pain management.^3,4^ Pharmacotherapies still rely on trial-and error-approaches in routine clinical practice, despite the potential of personalized pharmacotherapy.^5,6^ One promising strategy to address the variability in drug responses is pharmacogenetic (PGx) testing.^7^ By incorporating a patient’s genetic profile into medication analysis, PGx testing results may be used to tailor a safe and effective pharmacotherapy.^8^ This is particularly important for patients with complex conditions like chronic pain, where polypharmacy is common, medication changes are frequent, and the risk of drug-drug (DDI) and drug-gene interactions (DGI) is higher.^9^

Although research supports that PGx-guided therapies can optimize medication regimens, reduce adverse effects, and may offer economic benefits for selected patient populations (eg, in oncology, psychiatry, cardiology),^10-14^ the overall implementation in clinical practice is still limited. One major barrier in the implementation process is the lack of generic reimbursement frameworks due to limited evidence for economic and clinical utility.^15-17^ Specifically, it remains questionable whether the tangible and intangible efforts required for PGx testing are justified by its benefits, and for which patient populations this may be the case. Additionally, it is still unclear whether considering PGx testing results and implementing PGx-guided therapy would lead to measurable improvement in patient outcomes. As a result, stakeholders, including healthcare providers, policymakers, and insurers, remain skeptical of PGx and hesitant to invest in a necessary infrastructure for providing PGx testing, which would be required for a comprehensive integration into clinical decision-making.^18-20^

To provide evidence on the clinical utility of PGx testing, it is essential to focus on outcomes that matter most in the daily lives of affected patients,^21^ particularly those with complex and subjective conditions like chronic pain. For this, patient-reported outcomes (PROs) can be used to provide valuable insights into the real-world impact of an intervention by capturing the patient’s perception of their health state across various dimensions.^22-24^

The overall aim of this study was to assess the impact of PGx testing on real-world PROs in patients with chronic pain and to investigate differences between patients who received subsequent PGx-guided therapy and those who did not. Specifically, the first objective of this study was to assess changes in health-related quality of life (HRQoL) from pre-assessment to post-assessment in 3 groups: all patients who underwent PGx testing, those who underwent PGx testing and received subsequent PG-guided therapy, and those who underwent PGx testing but did not receive subsequent PGx-guided therapy. To complement this, our second objective was to evaluate changes in pain intensity, a disease specific PRO measure, from pre-assessment to-post-assessment within the same groups.

## Methods

### Study Population and Procedures

Our study population formed part of an observational pharmacist-led case series “Pharmacogenetic Testing of Patients with Unwanted Adverse Drug Reactions or Therapy Failure”,^25^ which was conducted according to the guidelines of the Declaration of Helsinki and approved by the Ethikkommission Nordwest- und Zentralschweiz (2019−01452), with a recent amendment approved on 31.10.2024. The study was reported after the STREGA checklist (Supplemental File: completed STREGA checklist).^26^

For the explorative pre-post analysis, patients were eligible, if they met the following inclusion criteria: (i) at least 18 years old, (ii) signed informed consent, (iii) diagnosed with a chronic pain condition, and (iv) suspected to have a drug-related problem (DRP, ie, adverse drug reactions [ADR] or therapy failure [TF]) under their current analgesic therapy including 1 or more drugs categorized by the Anatomical Therapeutic Chemical (ATC) classification as: Non-opioid analgesics (ATC third level: N02B); Non-steroidal anti-inflammatory drugs (NSAIDs, ATC third level: M01A); Opioid analgesics (ATC third level: N02A); Adjuvant analgesics under ATC first level: N (nervous system), for example, antidepressants (N06A), antiepileptics (N03A), psychostimulants (N06B), and anesthetics (N01A/N01B); Adjunct drugs: Drugs for peptic ulcer and gastro-esophageal reflux disease (ATC third level: A02B). Patients were excluded if they had insufficient German language skills or were not able to visit the study pharmacy in person.

These patients were referred to the study pharmacy by their medical specialist or general practitioner with all necessary documentation, including patient data, medical history, diagnoses, current medication list, details of the suspected drugs. Upon referral, verbal and written informed consent was provided. Subsequently, the study pharmacist performed a baseline assessment with the patient, starting with the pre-PGx assessment of PROs. Afterward, a buccal swab sample was collected from the patient for PGx panel testing. The genetic analysis including 95 variants in 30 genes (Supplemental Table 1) was performed by Stratipharm^®^ (humatrix AG, Pfungstadt, Germany) applying the TaqMan chemistry for Real Time polymerase chain reaction (PCR) to detect polymorphisms and gene duplications.

After a period of 2 to 3 weeks, the PGx panel test results were interpreted by the study pharmacist, supported by the clinical decision system of Stratipharm^®^ and additional research on PharmGKB (www.pharmgkb.org). A written PGx report was prepared, with the main genetic variations and thereon based medication recommendations individualized for each patient. The report included details on drugs suitable from a PGx perspective, current drugs requiring dosage adjustments, and drugs which should be avoided or replaced. In addition to the analysis of reactive DGIs, the report also included a table with preemptive recommendations for potential future medication. During a subsequent personal visit to the study pharmacy, the report was explained to the patient and sent to the referring physician and other relevant healthcare professionals (HCPs) of the patient, if requested.

The follow-up telephone interview was conducted with the patient 4 to 6 weeks after the written PGx report was explained to the patient and provided to the physician. The follow-up started with the post-PGx assessment of PROs. Afterward, changes in the patient’s medication were identified by comparing the medication list from study inclusion (pre-PGx assessment) with the current medication list (post-PGx assessment). The reasons for these changes and their initiation were discussed with the patient. If at least 1 medication change was found to have been made based on the reactive or preemptive recommendations of the PGx report, the patient was then classified as having received PGx-guided therapy. Conversely, if a patient had no medication changes between pre-PGx assessment and post-PGx assessment or if the medication changes were neither attributable to the reactive nor to the preemptive recommendations of the PGx report, the patient was classified as not having received PGx-guided therapy. In case of any uncertainties or questions that could not be clarified with the patient (eg, whether a medication change was based on the PGx report), they were addressed to the referring physician.

**Figure 1.**
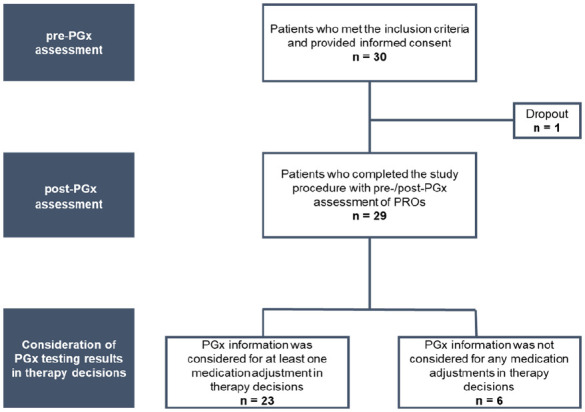
Study flowchart. A total of 30 patients met the inclusion criteria and provided informed consent. One patient dropped out, leaving 29 patients who completed the study procedure with pre-PGx and post-PGx assessment of PROs. Of these, the PGx report was considered in therapy decisions for 23 patients, while it was not considered for 6 patients. Abbreviations: n, number; PGx, pharmacogenetics; PROs, patient-reported outcomes.

The study flowchart is presented in Figure 1.

### Outcome Measures

Patient outcomes were assessed using the EuroQol 5-dimensional questionnaire (EQ-5D), which is a standardized and validated instrument developed by the EuroQol group to measure HRQoL.^27 [Bibr bibr28-11786329251356560]^ The EQ-5D-5L descriptive system evaluates health states across 5 dimensions: mobility, self-care, usual activities, pain/discomfort, and anxiety/depression, each rated on 5 levels of severity and encompasses all WHO-defined health domains from the International Association for the Study of Pain (IASP).^28^ Every health state is represented by a unique 5-digit code, where “11111” corresponds to perfect health and “55555” represents the worst possible health. Using a population-specific value set, these health state utilities were transformed into a single summary utility index, referred to as EQ index.^29-32^ The primary outcome of this study was the change in the EQ index from pre-PGx assessment to post-PGx assessment representing the overall HRQoL. Since a Swiss-specific value set is not available, the German value set was used for this calculation.^33^ The German value set allows EQ index scores from −0.611 to 1, where 1 represent the perfect health, 0 represents death, and values below 0 indicate health states perceived to be worse than death. A sensitivity analysis was conducted using the French value set, where EQ index scores range from −0.526 to 1.^34^

Pain intensity was assessed using the validated Numeric Rating Scale (NRS) adapted after McCaffery and Beebe (Supplemental File: NRS Pain Assessment Questionnaire Template German).^35,36^ Participants were asked to rate their pain on a scale from 0 to 10, where 0 indicates no pain, and 10 represents the worst imaginable pain. This pain assessment was evaluated across 3 scenarios: Current pain, highest pain in the past 24 hours, and lowest pain in the past 24 hours. A mean value was calculated based on these ratings. Therefore, the second outcome of this study was the change in NRS values from pre-PGx assessment to-post-PGx assessment representing an estimate of overall pain intensity. Both outcomes were assessed using an interviewer-administered version.

## Data Analysis

### Selection of MID

A threshold for the minimal important difference (MID) was selected to determine whether pre-PGx to post-PGx differences could be considered clinically relevant. The MID represents the smallest change in score within the domain of interest that patients perceive as important, whether beneficial or harmful, and that would lead a clinician to consider modifying the patient’s therapy management.^37^

The MID for changes in the EQ index reported in the literature ranges between 0.03 and 0.1. For our study on changes in EQ index scores pre-to-post PGx, calculated based on the German value set, we selected the MID of 0.083 (SD 0.022) after Henry et al.^38^ This study provides robust and patient-centered estimates of the MID for the EQ index, utilizing the standardized EQ-VT Version 2 valuation protocol. These estimates are tailored to country-specific value sets, such as the German one, incorporating societal preferences and health perceptions of the population.

In addition to the HRQoL based on the EQ index, pain intensity was evaluated as a condition specific PRO. To determine the MID for NRS, we selected a threshold based on a study with a population comparable to ours. The MID for changes in the NRS pre-to-post PGx was defined as a reduction of 1.74 points or 27.9%, respectively, as suggested by Farrar et al.^39^ This study was conducted in a large patient cohort with various chronic pain conditions.

### Statistics

Statistical analysis was conducted to evaluate changes in the first outcome (EQ index) and second outcome (NRS) from pre-to-post PGx. Prior to testing for group differences, data distribution was assessed using a Shapiro-Wilk test and visual inspection. For differences in non-normally distributed continuous data (EQ index) and ordinal data (NRS), the Wilcoxon signed-rank test was performed. In addition, a subgroup analysis was conducted by stratifying patients into those who received PGx-guided therapy and those who did not. To compare the proportion of patients who met the MID threshold and therefore achieved potentially clinically relevant improvements between these groups, a chi-square test was performed. Cohen’s d was calculated to quantify the effect size of changes in the EQ index from pre-to-post PGx within each group, along with the corresponding 95% confidence interval to provide an estimate of the precisions of the effect size. P values of .05 or lower were considered statistically significant, with no adjustment for multiple testing.

To visualize the findings, box plots were created illustrating the distribution of EQ index scores pre-PGx and post-PGx in those who received PGx-guided therapy and those who did not. Also, a scatter plot was generated to present the relationship between pre-PGx and post-PGx for the EQ index scores, considering the selected MID threshold for both groups.

Descriptive results are presented as absolute numbers (n) with percentages (%), mean with standard deviation (SD), or median with interquartile range (IQR). All analyses were conducted using Microsoft Office Professional Plus, Excel, version 16.0 (2016) and R, R Studio version 4.2.2 (2022).

## Results

### Characteristics

Between July 2023 and December 2024, a total of 29 eligible patients underwent the study procedures. The median age of the participants was 52 years [IQR 32-63], with a predominance of 22 female patients (76%). Seventeen participants (59%) were referred to the study by a medical specialist. Further characteristics are summarized in Supplemental Table 2.

All patients had conditions classified under ICD-10 category “R52.1/R52.2″: chronic pain. Fifteen patients (52%) had chronic pain associated with musculoskeletal and connective tissue diseases (ICD-10 category “M”), while 12 had primary pain diagnoses related to the nervous system (ICD-10 category “G,” 41%). Psychosomatic pain conditions, categorized under ICD-10 “F,” were present in 2 participants (7%).

Patients had a median of 5 [IQR 4-7] prescribed drugs in their current medication, with 19 patients experiencing polypharmacy (⩾5 drugs daily, 83%). The first patient visit lasted a median of 30 minutes [IQR 25-35], while the second visit had a median of 25 minutes [20-30]. The interpretation of the patients’ PGx testing results, as well as the preparation of the PGx report, required an average of 120 minutes per patient [IQR 110-180]. The telephone follow-up (post-PGx assessment) was conducted with a median of 49 days [40-62] after the second visit and a median of 98 days [71-112] after the first patient visit (pre-PGx assessment).

Overall, the 29 patients had in total 157 suspicions for a DGI, based on a DRP. Each patient had a median of 5 suspicions for a DGI [IQR 4-7]. The most frequently suspected drugs were antidepressants (ATC third level: N06A) and non-opioid analgesics (ATC third level: N02B) with each 36 suspicions (23%), followed by anti-inflammatory drugs (ATC third level: M01A) with 24 suspicions (15%) and opioids (ATC third level: N02A) with 14 suspicions (9%).

After PGx analysis, it was possible to confirm 60 DGI suspicions (38%), based on at least 1 identified genetic variation, with a median of 2 confirmed DGI per patient [IQR 1-3]. Among the confirmed DGIs, antidepressants (ATC third level: N06A) were the most commonly involved with 19 confirmations (31%), followed by opioids (ATC third level: N02A) with 11 confirmations (18%), drugs used for the treatment of peptic ulcers and gastroesophageal reflux disease (ATC third level: A02B) with 8 confirmations (13%) and anti-inflammatory drugs (ATC third level: M01A) with 6 confirmations (11%). The majority of the DGI (57%) were identified based on variations in *CYP2D6*, *CYP2C19*, and *CYP2C9*. Those genotype-predicted phenotypes corresponded to the frequencies of the European Caucasian population.^40^

In the follow-up, it was possible to detect a total of 49 medication changes in 23 patients (79%) that were in line with the recommendations in the PGx report, with a median of 2 medication changes per patient [IQR 1-2]. The most common medication change was the initiation of a new drug in 27 cases (55%), followed by 15 discontinuation of a drug therapy (31%), and 4 dosage adaptations (8%).

### EQ Index

The 5-digit codes ranged from the best score “1111” (EQ index: 1) to “42555” (EQ index: −0.366). A wide distribution was observed with mean EQ index scores of 0.379 (SD 0.420) pre-PGx and 0.697 (SD 0.307) post-PGx (Table 1). The difference between pre-PGx and post-PGx EQ index scores was statistically significant (*P* < .001). The overall effect size of the change from pre-PGx to post-PGx can be described as large based on Cohen’s d, with −0.84 (95% CI: −1.22, −0.55). Changes between pre-PGx and post-PGx testing for the single dimensions are illustrated in Supplemental Figure 1, with the corresponding numerical results summarized in Supplemental Table 3. Notably, in the post-PGx assessment an increase in the level of severity “no problems” was observed across all dimensions, while “severe problems” decreased overall. The individual 5-digit codes and the resulting EQ index scores calculated with the German value set can be found in Supplemental Figure 2. The sensitivity analysis with the French value set led EQ index scores comparable to those derived with the German value set, with a tendency toward higher EQ index scores based on a mean difference between both value sets of 0.04 (SD 0.04), shown in Supplemental Figure 3.

**Table 1.** Descriptive and statistical analysis of EQ index scores pre-PGx and post-PGx assessment.

Group	n	EQ index pre-PGx mean (SD)	EQ index post-PGx mean (SD)	EQ index pre-to-post PGx Δ mean (SD)	Effect size Cohen’s d (95% CI)	*P*-value
All patients	29	0.379 (0.420)	0.697 (0.307)	0.317 (0.352)	−0.84 (−1.22, −0.55)	<.001^a^
PGx-guided therapy	23	0.349 (0.429)	0.729 (0.262)	0.380 (0.357)	-1.00 (−1.56, −0.64)	<.001^a^
Non-PGx-guided therapy	6	0.497 (0.396)	0.572 (0.449)	0.074 (0.204)	−0.17 (−1.01, 0.09)	NS: .589^a^

Abbreviations: EQ, EuroQol; NS, not significant; PGx, pharmacogenetics; SD, standard deviation.

a Wilcoxon signed-rank test.

**Figure 2.**
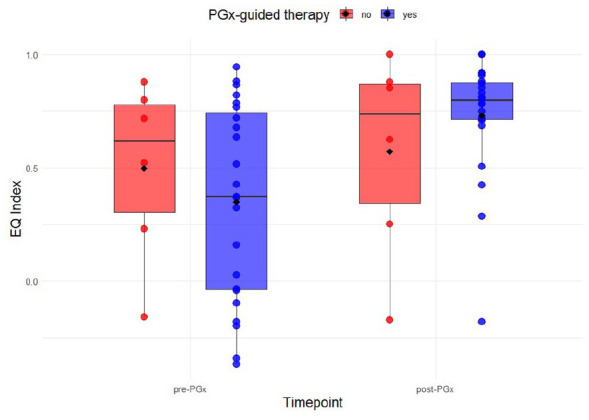
EQ index score distribution pre-PGx and post-PGx stratified by PGx-guided therapy. The boxplots illustrate the distribution of EQ index scores pre-PGx and post-PGx testing, classified by whether patients received PGx-guided therapy (blue; right side) or not (red; left side). The median value (black line), the mean value (diamond), and individual patient scores (dots) are displayed for each group. Abbreviations: EQ, EuroQol; PGx, pharmacogenetics.

Across all 29 patients, 23 were stratified as having received PGx-guided therapy after PGx testing, while 6 were classified as not having received PGx-guided therapy. In the PGx-guided therapy group, the change in the EQ index scores was statistically significant, while it was not significant in the non-PGx-guided therapy group (Table 1). The overall effect from pre-PGx to post-PGx was very large in the PGx-guided therapy group based on Cohen’s d with -1.00 (95% CI: −1.56, −0.64), whereas the non-PGx-guided therapy group showed a small effect with −0.17 (95% CI: −1.01, 0.09). Patients in the non-PGx-guided therapy group had higher baseline EQ index scores before PGx testing compared to those in the PGx-guided therapy group. However, after PGx testing, their EQ index scores showed only a minimal increase (Figure 2).

With respect to clinical relevance, the EQ index change of 19 patients met the selected MID criterion of 0.083 (SD 0.022),^38^ while 10 did not. Of the 19 patients who met the MID criterion, 18 had undergone PGx-guided therapy. Among the 10 patients whose EQ index scores did not reach the selected MID, 5 had not received PGx-guided therapy. The difference between the PGx-guided and non-PGx-guided therapy groups in terms of reaching the MID threshold was statistically significant (*P* = .01).

**Figure 3.**
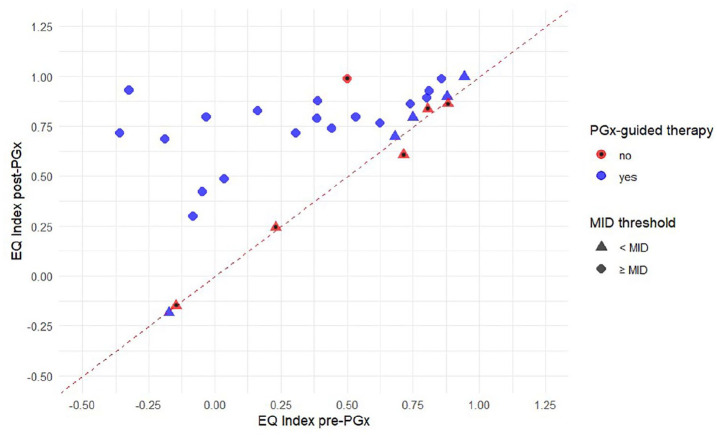
Scatter plot of all patients’ EQ index score pre-PGx and post-PGx in consideration of the MID threshold and PGx-guided therapy. Each point/triangle represents an individual patient. The red line (*y* = *x*) indicates no change in EQ index scores. Point/triangle above the line represent an improvement in EQ index score, while those below the line indicate a decrease. Red colored points/triangles with a dot represent patients without PGx-guided therapy, while blue colored without a dot represent those with PGx-guided therapy. Abbreviations: EQ, EuroQol; MID, minimal important difference; PGx, pharmacogenetics.

The relationship between EQ index scores pre-PGx and post-PGx, considering the MID threshold and PGx-guided therapy, is illustrated in Figure 3.

### NRS

Across all 29 patients, a reduction in pain intensity was observed in all measured categories: current pain intensity, highest pain intensity, lowest pain intensity, and mean pain intensity per patient between pre-PGx and post-PGx assessment. The greatest mean reduction was observed in the highest pain intensity, closely followed by the lowest pain intensity. The mean differences were statistically significant across all categories (Table 2). However, none of the differences between pre-PGx and post-PGx met the predefined MID threshold of a reduction of 1.74.

**Table 2.** Descriptive and statistical analysis of NRS scores pre-PGx and post-PGx assessment.

Group	n	Current		Highest		Lowest		Mean per patient	
		Pre-to-post PGx Δ mean (SD)	*P*-value	Pre-to-post PGx Δ mean (SD)	*P*-value	Pre-to-post PGx Δ mean (SD)	*P*-value	Pre-to-post PGx Δ mean (SD)	*P*-value
All patients	29	−1.37 (1.63)	<.001	−1.72 (1.81)	<.001	−1.62 (1.66)	<.001	−1.58 (1.21)	<.001
PGx-guided therapy	23	−1.41 (1.37)	.001	−2.00 (1.57)	<.001	−2.04 (1.58)	.005	−1.82 (1.01)	<.001
Non-PGx-guided therapy	6	−1.25 (2.56)	NS: .434^a^	−0.67 (2.42)	NS: .530^a^	0.00 (0.63)	NS: 1.000^a^	−0.63 (1.55)	NS: .563^a^

Abbreviations: n, number; NRS, numeric rating scale; NS, not significant; PGx, pharmacogenetics; SD, standard deviation.

a Wilcoxon signed-rank test.

**Figure 4.**
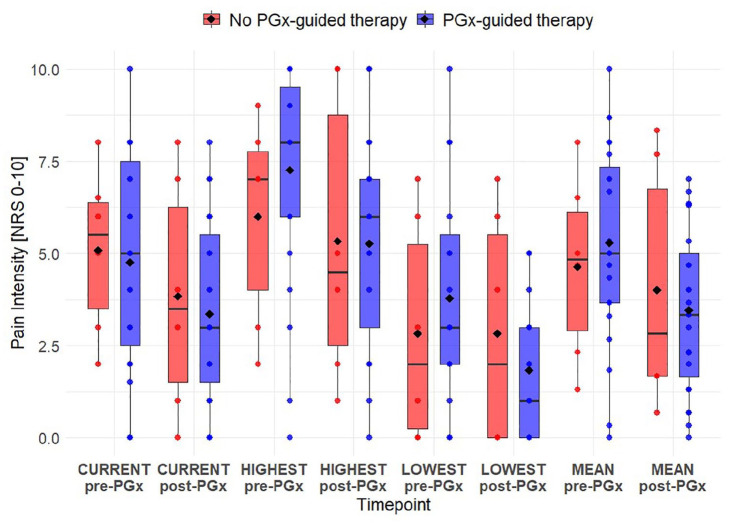
Pain intensity distribution pre-PGx and post-PGx in patients stratified by PGx-guided therapy. The boxplots illustrate the distribution of pain intensity scores on the NRS scale [0-10] pre-PGx and post-PGx, classified by whether patients received PGx-guided therapy (blue; right side) or not (red; left side). The median value (black line), the mean value (diamond), and individual patient scores (dots) are displayed for each group. Abbreviations: EQ, EuroQol; NRS, numeric rating scale, PGx, pharmacogenetics.

When stratifying patients into those who received PGx-guided therapy, and those who did not, differences in NRS score changes were observed (Figure 4). Among the 23 patients who received PGx-guided therapy, a decrease in pain intensity was assessed across all categories from pre-PGx to post-PGx. In this group, the differences between pre-PGx assessment and post-PGx assessment were statistically significant for all categories of pain intensity (Table 2). In contrast, among the 6 patients who did not receive PGx-guided therapy, no statistically significant differences were observed in any category of pain intensity (Table 2). In addition, in this group, the lowest pain intensity remained stable form pre-PGx to post-PGx (Figure 4). Furthermore, in the non-PGx-guided therapy group, none of the reductions met the selected MID threshold of 1.74, whereas in the PGx-guided therapy group, the reductions in the highest, lowest, and mean pain intensity per patient exceeded the MID threshold.

## Discussion

### Improvement of PROs After PGx

This exploratory study suggests that PGx-guided therapy may lead to measurable and clinically relevant improvements in PROs in chronic pain patients. While minor changes were also observed in the non-PGx-guided therapy group, they did not reach statistical significance or the predefined MID thresholds indicating clinical relevance. In contrast, the PGx-guided therapy group showed statistically significant improvements that were also potentially clinically relevant. This observation was consistent across both HRQoL and pain intensity, and underscores the importance of implementing pharmaceutical recommendations after PGx testing to provide real-world benefits for patients with long-term conditions, such as chronic pain.

In general, our observations align with previous literature demonstrating that PGx-based medication adjustments can positively impact HRQoL by optimizing drug efficacy and minimizing ADR.^11,14^ To our knowledge, no prior study has specifically assessed EQ index scores in a comparable study population of chronic pain patients, making this study a contribution to the growing evidence supporting the relevance of PGx-guided therapies. Interestingly, beyond the average improvement in EQ index, we also observed a notable decrease in variability (ie, SD and IQR) under the PGx-guided therapy. This may suggest that PGx-guided therapy not only improves individual outcomes, but also contributes to greater consistency across patients by reducing overall variability in drug response. Such homogenization may be particularly relevant in clinical practice, as it can support more predictable treatment effects. This observation could inform future population-based research on PGx interventions. Beyond the EQ index, significant pre-to-post differences were observed in the second outcome, pain intensity, across all 29 patients. This observations also align with existing literature indicating that PGx-informed pain management can lead to better patient outcomes.^12,41^

#### PGx Testing and Counseling in Chronic Pain Patients

In our study, 79% of the study population had at least 1 medication change based on the provided PGx report. This represents a high implementation rate of PGx-guided medication adjustments compared to other studies, where rates ranged between 62% and 69.9%.^10,42,43^ The high implementation rate might be attributed to the selected study population, consisting of chronic pain patients, who represent a suitable target group for PGx-guided therapy as analgesic pharmacotherapies often involve a wide range of PGx-actionable drugs.^44-47^ There are already studies suggesting that PGx testing should be integrated into pain management, as the identification of genetic variations can help to predict individual drug responses, which is particularly important for the complex and subjective nature of chronic pain.^48,49^ In addition to medication analysis, which included the reactive identification of DGIs, we also provided preemptive PGx-based medication recommendations, allowing for a more personalized selection of opioids, non-opioid analgesics, adjuvant, and adjunct drugs in the future.^50^ This comprehensive approach may be particularly beneficial for this patient population as 83% of our study sample experienced polypharmacy and were therefore at high risk for DDIs, DGIs and even drug-drug-gene interactions (DDGIs).

Since minor changes in HRQoL and pain intensity were also observed in the non-PGx-guided therapy group, we suspect that additional factors may have influenced the PROs. In particular, the extensive consultation and counseling may also have contributed to PRO improvements. All patients received a median of 55 minutes of direct consultation across 2 visits. Beyond these visits, an additional median of 120 minutes per patient was spent on the interpretation of PGx results and preparation of the individualized reports with preemptive recommendations. During the consultations, patients engaged in discussions about their medications, potential side effects, and diagnostic considerations. The PGx report was also shared with the referring physician, and other HCPs involved in the patient’s care, fostering interdisciplinary collaboration. This approach likely contributed to the improved outcomes, as shared decision-making has been shown to enhance patient satisfaction.^22,51^ In addition, chronic pain patients frequently experience drug changes, which can lead to uncertainty and frustration.^52,53^ The combination of PGx testing and structured counseling may have addressed these concerns by providing not only clarity on future drug choices, but also insights why certain drugs have failed in the past. This aspect might include an important psychological component and serve as a source of relief for patients.^54,55^ In addition, patients experiencing more severe pain may be more likely to seek medical attention and may be more receptive to PGx testing and subsequent therapy changes. This might confirm the observation that patients undergoing PGx-guided therapy had higher baseline scores for the highest, lowest and average pain intensity than those who did not.

Besides the PGx intervention itself, it is important to consider that the natural variability of pain fluctuations over time may have also contributed to the observed pre-to-post differences.^56^ Chronic pain conditions can change independently of any intervention due to their dynamic nature.^57^ Furthermore, pain perception varies between individuals and can be influenced by patient characteristics such as sex. It should be noted that female sex has been associated with increased pain sensitivity and a higher risk of TF from analgesic therapy.^58-61^ This may also be reflected in our study population, which included 22 female participants and showed a high prevalence of DRP.

Focusing on the real-world application of PGx, our study framework provides a foundation for establishing a reliable standard of procedure in clinical practice. However, the time investment in our study does not realistically reflect the time resources available in routine clinical settings. For the real-world implementation of PGx, it is essential to ensure that sufficient resources are allocated for PGx testing and subsequent counseling, to ensure the effort invested in these procedures can translate into clinical benefit for the patient.^62,63^

#### Limitations and Future Directions

This study has several limitations that should be considered. First, while efforts were made to standardize pre-to-post assessment of PROs, differences in data collection methods (pre-PGx in-person vs post-PGx as telephone-based interviews) may have introduced a response bias. However, existing literature suggests that telephone respondents are more likely to express dissatisfaction,^64^ which may lead to more honest responses rather than favoring more favorable responses in the post-PGx assessment. Second, from a methodological perspective, this study assessed health state utilities through PRO assessment, demonstrating potentially clinically relevant trends toward outcome improvements in the PGx-guided therapy group. The non-PGx-guided therapy group was not designed as a control group and should rather be seen as an opportunity for observational comparison that emerged during the study. This means we cannot reliably determine whether the observed pre-to-post changes were solely due to PGx-based medication changes. In addition, the sample size was small and limits the statistical power of the study. Due to the exploratory nature of the study, no power analysis was conducted. Statistical findings regarding pre-to-post differences might differ in a larger study population, particularly given the unequal group sizes between patients who received PGx-guided therapy and those who did not. Moreover, the study was exploratory with multiple statistical tests, without a correction for multiple testing, which increases the risk of type I errors.^65,66^ In this context, it is also important to note that the small sample size in the non-PGx-guided therapy group (n = 6) limited the ability to detect statistically significant effects. While this may have contributed to the non-significant findings observed in that group, we chose not to conduct sample size calculations for future studies, as such estimates based on observed effects of small samples may be unreliable. Instead, these limitations highlight the need for adequately powered, prospective studies to confirm our exploratory findings.

Third, due to the absence of a randomized control group, no causal conclusions can be drawn from our results. The observed associations should be interpreted as exploratory and hypothesis-generating. Lastly, the study did not incorporate cost calculations and was therefore not a pharmacoeconomic study. However, our study findings could serve as a foundation for future pharmacoeconomic studies investigating whether PGx interventions are clinically relevant and financially viable in the long term. Such studies are essential in the overall implementation process and will be critical in informing policy discussion regarding PGx reimbursement.

## Conclusion

This explorative study aimed to investigate the association between PGx testing, HRQoL, and pain intensity in chronic pain patients, and to assess potential differences between patients who received subsequent PGx-guided therapy and those who did not. We observed that EQ index scores significantly improved after PGx testing, reflecting enhancements in overall HRQoL, as well as in NRS, indicating a reduction in pain intensity. The stratification between patients with PGx-guided therapy and those without suggests that the overall pre-to-post differences in PROs might be attributed to PGx-guided therapy, as potentially clinically relevant improvements were observed for both outcomes within this group. Based on these observations, we hypothesize that PGx testing may only lead to relevant improvements in outcome if PGx-based medication recommendations are actively integrated into therapy decisions. By combining a patient-centered methodology with a highly relevant PGx target population, the results of this study indicate that a broader clinical adoption of PGx testing in routine practice may be warranted. They may serve as a foundation for future clinical and economic studies, which should be conducted on a large scale and with randomized designs to assess the long-term clinical impact and economic feasibility of PGx-guided therapy. Ultimately, this could inform policy and reimbursement decisions within healthcare systems.

## Supplemental Material

sj-docx-1-his-10.1177_11786329251356560 – Supplemental material for Association of Pharmacogenotyping and Patient-Reported Outcomes in Chronic Pain Management Supplemental material, sj-docx-1-his-10.1177_11786329251356560 for Association of Pharmacogenotyping and Patient-Reported Outcomes in Chronic Pain Management by Anna Bollinger, Kurt E. Hersberger, Julia Gianora, Isabelle O. Urdieux, Henriette E. Meyer zu Schwabedissen, Nikki Rommers, Matthias Schwenkglenks, Céline K. Stäuble and Samuel S. Allemann in Health Services Insights

sj-docx-2-his-10.1177_11786329251356560 – Supplemental material for Association of Pharmacogenotyping and Patient-Reported Outcomes in Chronic Pain Management Supplemental material, sj-docx-2-his-10.1177_11786329251356560 for Association of Pharmacogenotyping and Patient-Reported Outcomes in Chronic Pain Management by Anna Bollinger, Kurt E. Hersberger, Julia Gianora, Isabelle O. Urdieux, Henriette E. Meyer zu Schwabedissen, Nikki Rommers, Matthias Schwenkglenks, Céline K. Stäuble and Samuel S. Allemann in Health Services Insights

sj-docx-3-his-10.1177_11786329251356560 – Supplemental material for Association of Pharmacogenotyping and Patient-Reported Outcomes in Chronic Pain Management Supplemental material, sj-docx-3-his-10.1177_11786329251356560 for Association of Pharmacogenotyping and Patient-Reported Outcomes in Chronic Pain Management by Anna Bollinger, Kurt E. Hersberger, Julia Gianora, Isabelle O. Urdieux, Henriette E. Meyer zu Schwabedissen, Nikki Rommers, Matthias Schwenkglenks, Céline K. Stäuble and Samuel S. Allemann in Health Services Insights

sj-docx-4-his-10.1177_11786329251356560 – Supplemental material for Association of Pharmacogenotyping and Patient-Reported Outcomes in Chronic Pain Management Supplemental material, sj-docx-4-his-10.1177_11786329251356560 for Association of Pharmacogenotyping and Patient-Reported Outcomes in Chronic Pain Management by Anna Bollinger, Kurt E. Hersberger, Julia Gianora, Isabelle O. Urdieux, Henriette E. Meyer zu Schwabedissen, Nikki Rommers, Matthias Schwenkglenks, Céline K. Stäuble and Samuel S. Allemann in Health Services Insights

sj-docx-5-his-10.1177_11786329251356560 – Supplemental material for Association of Pharmacogenotyping and Patient-Reported Outcomes in Chronic Pain Management Supplemental material, sj-docx-5-his-10.1177_11786329251356560 for Association of Pharmacogenotyping and Patient-Reported Outcomes in Chronic Pain Management by Anna Bollinger, Kurt E. Hersberger, Julia Gianora, Isabelle O. Urdieux, Henriette E. Meyer zu Schwabedissen, Nikki Rommers, Matthias Schwenkglenks, Céline K. Stäuble and Samuel S. Allemann in Health Services Insights

sj-docx-6-his-10.1177_11786329251356560 – Supplemental material for Association of Pharmacogenotyping and Patient-Reported Outcomes in Chronic Pain Management Supplemental material, sj-docx-6-his-10.1177_11786329251356560 for Association of Pharmacogenotyping and Patient-Reported Outcomes in Chronic Pain Management by Anna Bollinger, Kurt E. Hersberger, Julia Gianora, Isabelle O. Urdieux, Henriette E. Meyer zu Schwabedissen, Nikki Rommers, Matthias Schwenkglenks, Céline K. Stäuble and Samuel S. Allemann in Health Services Insights
