# Supplementary material for: Association of Pharmacogenotyping and Patient-Reported Outcomes in Chronic Pain Management
Source: Health Serv Insights. 2025 Jul 12;18:11786329251356560. doi: 10.1177/11786329251356560 (PMC12255864; doi:10.1177/11786329251356560)
Supplement: sj-docx-1-his-10.1177_11786329251356560 – Supplemental material for Association of Pharmacogenotyping and Patient-Reported Outcomes in Chronic Pain Management [file sj-docx-1-his-10.1177_11786329251356560.docx]

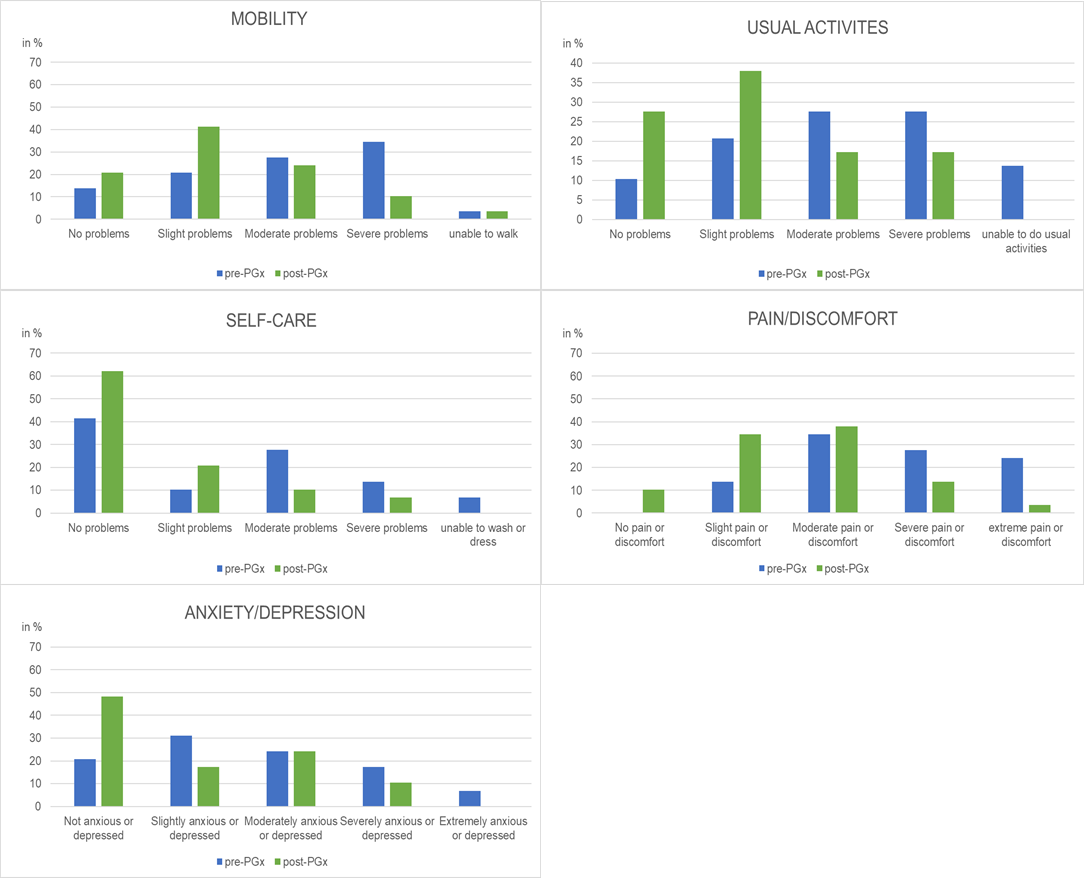


**Supplementary Figure 1. Changes in the single dimensions of the EQ-5D-5L pre-to-post PGx in all patients.**  Description: The bar charts illustrate the percentage distribution of different health states across mobility, usual activities, self-care, pain/discomfort, anxiety/depression, before (blue) and after (green) PGx. Abbreviation: PGx, pharmacogenetics**.**
