# Supplementary material for: Association of Pharmacogenotyping and Patient-Reported Outcomes in Chronic Pain Management
Source: Health Serv Insights. 2025 Jul 12;18:11786329251356560. doi: 10.1177/11786329251356560 (PMC12255864; doi:10.1177/11786329251356560)
Supplement: sj-docx-2-his-10.1177_11786329251356560 – Supplemental material for Association of Pharmacogenotyping and Patient-Reported Outcomes in Chronic Pain Management [file sj-docx-2-his-10.1177_11786329251356560.docx]

**Supplementary Figure 2. Individuals’ 5-digit-code with corresponding EQ index.**

Abbreviations: EQ, EuroQol; PGx, pharmacogenetics.
