# Supplementary material for: Association of Pharmacogenotyping and Patient-Reported Outcomes in Chronic Pain Management
Source: Health Serv Insights. 2025 Jul 12;18:11786329251356560. doi: 10.1177/11786329251356560 (PMC12255864; doi:10.1177/11786329251356560)
Supplement: sj-docx-4-his-10.1177_11786329251356560 – Supplemental material for Association of Pharmacogenotyping and Patient-Reported Outcomes in Chronic Pain Management [file sj-docx-4-his-10.1177_11786329251356560.docx]

**Supplementary Table 1.** Stratipharm^®^ (humatrix AG, Pfungstadt, Germany) PGx panel test (single nucleotide polymorphisms and annotations).

| **Gene** | **Chromosome** | **Annotation** | **Position** | **Amino acid replacement** |
| --- | --- | --- | --- | --- |
| ABCB1 | Chromosom 7q21.12 | rs1045642 | NM_000927.4:c.3435T>C | I1145I |
| ABCB1 | Chromosom 7q21.12 | rs1128503 | NM_000927.4:c.1236T>C | G412G |
| ABCB1 | Chromosom 7q21.12 | rs2032582 | NM_000927.4:c.2677G>A | A893T |
| ABCB1 | Chromosom 7q21.12 | rs2032582 | NM_000927.4:c.2677G>T | A893S |
| ABCB1 | Chromosom 7q21.12 | rs2032583 | NM_000927.4:c.2685+49T>C | - |
| ABCG2 | Chromosom 4q22-q23 | rs2231142 | NM_004827.2:c.421C>A | Q141K |
| ABCG2 | Chromosom 4q22-q23 | rs13120400 | NM_004827.2:c.1194+928A>G | - |
| ABCG2 | Chromosom 4q22-q23 | rs17731538 | NC_000004.11:g.89055379G>A | - |
| ADRB1 | Chromosom 10q24-q26 | rs1801252 | NM_000684.2:c.145A>G | S49G |
| ADRB1 | Chromosom 10q24-q26 | rs1801253 | NM_000684.2:c.1165G>C | G389R |
| ADRB2 | Chromosom 5q31-q32 | rs1042713 | NT_029289.11:g.9369367G>A | G16R |
| ADRB2 | Chromosom 5q31-q32 | rs1042714 | NC_000005.9:g.148206473G>C | E27Q |
| COMT | Chromosom 22q11.21 | rs4680 | NM_000754.3:c.472G>A | V158M |
| COMT | Chromosom 22q11.21 | rs165599 | NM_000754.3:c.*522G>A | - |
| COMT | Chromosom 22q11.21 | rs4646316 | NM_000754.3:c.615+310C>T | - |
| COMT | Chromosom 22q11.21 | rs9332377 | NM_000754.3:c.616-367C>T | - |
| COQ2 | Chromosom 4q21.23 | rs4693075 | NC_000004.11:g.84192168G>C | - |
| COQ2 | Chromosom 4q21.23 | rs6535454 | NM_015697.7:c.894T>C | D298D |
| CYP1A2 | Chromosom 15q24.1 | rs2069514 | NC_000015.9:g.75038220G>A | - |
| CYP1A2 | Chromosom 15q24.1 | rs762551 | NC_000015.9:g.75041917C>A | - |
| CYP2B6 | Chromosom 19q13.2 | rs8192709 | NM_000767.4:c.64C>T | R22C |
| CYP2B6 | Chromosom 19q13.2 | rs28399499 | NM_000767.4:c.983T>C | I328T |
| CYP2B6 | Chromosom 19q13.2 | rs3745274 | NM_000767.4:c.516G>T | Q172H |
| CYP2C8 | Chromosom 10q24.1 | rs10509681 | NM_000770.3:c.1196A>G | K399R |
| CYP2C8 | Chromosom 10q24.1 | rs11572080 | NM_000770.3:c.416G>A | R139K |
| CYP2C8 | Chromosom 10q24.1 | rs1934951 | NG_007972.1:g.35707G>A | - |
| CYP2C9 | Chromosom 10q24.1 | rs1799853 | NM_000771.3:c.430C>T | R144C |
| CYP2C9 | Chromosom 10q24.1 | rs1057910 | NM_000771.3:c.1075A>C | I359L |
| CYP2C9 | Chromosom 10q24.1 | rs9332131 | NM_000771.3:c.817delA | K273X |
| CYP2C9 | Chromosom 10q24.1 | rs7900194 | NM_000771.3:c.449G>A | R150H |
| CYP2C9 | Chromosom 10q24.1 | rs28371685 | NM_000771.3:c.1003C>T | R335W |
| CYP2C19 | Chromosom 10q24 | rs4244285 | NM_000769.1:c.681G>A | - |
| CYP2C19 | Chromosom 10q24 | rs4986893 | NM_000769.1:c.636G>A | W212X |
| CYP2C19 | Chromosom 10q24 | rs12248560 | NG_008384.1:g.4195C>T | - |
| CYP2C19 | Chromosom 10q24 | rs28399504 | NM_000769.1:c.1A>G | M1V |
| CYP2D6 | Chromosom 22q13.1 | - | copy number variation | - |
| CYP2D6 | Chromosom 22q13.1 | rs35742686 | NM_000106.4:c.775delA | - |
| CYP2D6 | Chromosom 22q13.1 | rs3892097 | NM_000106.4:c.506-1G>A | - |
| CYP2D6 | Chromosom 22q13.1 | rs5030655 | NM_000106.4:c.454delT | - |
| CYP2D6 | Chromosom 22q13.1 | rs5030867 | NM_000106.4:c.971A>C | H324P |
| CYP2D6 | Chromosom 22q13.1 | rs5030865 | NM_000106.4:c.505G>T | G169X |
| CYP2D6 | Chromosom 22q13.1 | rs5030865 | NM_000106.4:c.505G>A | G169R |
| CYP2D6 | Chromosom 22q13.1 | rs5030656 | NM_000106.5:c.841_843delAAG | K281del |
| CYP2D6 | Chromosom 22q13.1 | rs1065852 | NM_000106.4:c.100C>T | P34S |
| CYP2D6 | Chromosom 22q13.1 | rs201377835 | NM_000106.5:c.181-1G>C | - |
| CYP2D6 | Chromosom 22q13.1 | rs28371706 | NM_000106.4:c.320C>T | T107I |
| CYP2D6 | Chromosom 22q13.1 | rs59421388 | NM_000106.4:c.1012G>A | V338M |
| CYP2D6 | Chromosom 22q13.1 | rs28371725 | NM_000106.4:c.985+39G>A | - |
| CYP3A4 | Chromosom 7q21.1 | rs2740574 | NG_000004.3:g.135607G>A | - |
| CYP3A4 | Chromosom 7q21.1 | rs2242480 | NM_017460.5:c.1026+12G>A | - |
| CYP3A5 | Chromosom 7q21.1 | rs776746 | NM_000777.3:c.219-237G>A | - |
| DPYD | Chromosom 1p22 | rs3918290 | NM_000110.3:c.1905+1G>A | - |
| DPYD | Chromosom 1p22 | rs72549303 | NM_000110.3:c.1898delC | - |
| DPYD | Chromosom 1p22 | rs72549309 | NM_000110.3:c.298delTinsTCAT | - |
| DPYD | Chromosom 1p22 | rs55886062 | NM_000110.3:c.1679T>G | I560S |
| DPYD | Chromosom 1p22 | rs67376798 | NM_000110.3:c.2846A>T | D949V |
| DPYD | Chromosom 1p22 | rs2297595 | NM_000110.3:c.496A>G | M166V |
| GNB3 | Chromosom 12p13 | rs5443 | NM_002075.2:c.825C>T | S275S |
| GSTP1 | Chromosom 11q13.2 | rs1695 | NM_000852.3:c.313A>G | I105V |
| HLA-A | Chromosom 6p21.3 | rs1061235 | NM_002116.7:c.*66A>T | - |
| HLA-A | Chromosom 6p21.3 | rs1633021 | NC_000006.12:g.29779092T>C | - |
| HLA-B | Chromosom 6p21.3 | rs3909184 | NM_005803.2:c.724-507C>G | - |
| HLA-B | Chromosom 6p21.3 | rs2395029 | NM_006674.3:c.*568T>G | - |
| HLA-B | Chromosom 6p21.3 | rs2844682 | NC_000006.11:g.30946148G>A | - |
| HMGCR | Chromosom 5q13.3-q14 | rs17238540 | NM_000859.2:c.2457+117T>G | - |
| HMGCR | Chromosom 5q13.3-q14 | rs17244841 | NM_000859.2:c.451-174A>T | - |
| HTR2A | Chromosom 13q14-q21 | rs6311 | NC_000013.10:g.47471478C>T | - |
| HTR2A | Chromosom 13q14-q21 | rs6313 | NM_000621.3:c.102C>T | S34S |
| HTR2A | Chromosom 13q14-q21 | rs7997012 | NM_000621.3:c.614-2211T>C | - |
| HTR2A | Chromosom 13q14-q21 | rs9316233 | NC_000013.10:g.47433355C>G | - |
| HTR2A | Chromosom 13q14-q21 | rs6314 | NC_000013.10:g.47409034G>A | H368Y |
| IFNL3 | Chromosom 19q13.13 | rs8099917 | NC_000019.9:g.39743165T>G | - |
| IFNL3 | Chromosom 19q13.13 | rs12979860 | NC_000019.9:g.39738787C>T | - |
| ITPA | Chromosom 20p | rs1127354 | NM_181493.1:c.43C>A | P32T |
| MT-RNR1 | mitochondrial DNA | rs267606617 | NC_012920.1:m.1555A>G | - |
| NAT2 | Chromosom 8p22 | rs1801280 | NM_000015.2:c.341T>C | I114T |
| NAT2 | Chromosom 8p22 | rs1799930 | NM_000015.2:c.590G>A | R197Q |
| NAT2 | Chromosom 8p22 | rs1799931 | NM_000015.2:c.857G>A | G286E |
| OPRM1 | Chromosom 6q24-q25 | rs1799971 | NM_000914.3:c.118A>G | N40D |
| SLC19A1 | Chromosom 21q22.3 | rs1051266 | NM_194255.1:c.80A>G | H27R |
| SLCO1B1 | Chromosom 12p12 | rs4149056 | NM_006446.4:c.521T>C | V174A |
| SLCO1B1 | Chromosom 12p12 | rs11045819 | NM_006446.4:c.463C>A | P155T |
| SLCO1B1 | Chromosom 12p12 | rs2306283 | NM_006446.4:c.388A>G | N130D |
| SLCO1B1 | Chromosom 12p12 | rs4149015 | NG_011745.1:g.4195G>A | - |
| TPMT | Chromosom 6p22.3 | rs1800462 | NM_000367.2:c.238G>C | A80P |
| TPMT | Chromosom 6p22.3 | rs1800460 | NM_000367.2:c.460G>A | A154T |
| TPMT | Chromosom 6p22.3 | rs1142345 | NM_000367.2:c.719A>G | Y240C |
| TPMT | Chromosom 6p22.3 | rs1800584 | NM_000367.2:c.626-1G>A | - |
| TPMT | Chromosom 6p22.3 | rs12201199 | NM_000367.2:c.419+94T>A | - |
| VKORC1 | Chromosom 16p11.2 | rs9923231 | NC_000016.9:g.31107689C>T | - |
| VKORC1 | Chromosom 16p11.2 | rs7294 | NM_024006.4:c.*134G>A | - |
| VKORC1 | Chromosom 16p11.2 | rs17708472 | NM_024006.4:c.173+525C>T | - |
| VKORC1 | Chromosom 16p11.2 | rs2359612 | NM_024006.4:c.283+837T>C | - |
| VKORC1 | Chromosom 16p11.2 | rs8050894 | NM_024006.4:c.283+124G>C | - |
| VKORC1 | Chromosom 16p11.2 | rs9934438 | NM_024006.4:c.174-136C>T | - |
