# Supplementary material for: Association of Pharmacogenotyping and Patient-Reported Outcomes in Chronic Pain Management
Source: Health Serv Insights. 2025 Jul 12;18:11786329251356560. doi: 10.1177/11786329251356560 (PMC12255864; doi:10.1177/11786329251356560)
Supplement: sj-docx-5-his-10.1177_11786329251356560 – Supplemental material for Association of Pharmacogenotyping and Patient-Reported Outcomes in Chronic Pain Management [file sj-docx-5-his-10.1177_11786329251356560.docx]

**Supplementary Table 2**. Patients‘ characteristics.

| **Characteristic** | **Category** | **Value** |
| --- | --- | --- |
| Female | PGx-guided therapy  Non-PGx guided therapy | 19/23  3/6 |
| Male | PGx-guided therapy  Non-PGx guided therapy | 2/23  3/6 |
| Younger adults (18-44 years) | PGx-guided therapy  Non-PGx guided therapy | 7/10  3/10 |
| Middle-aged adults (45-64 years) | PGx-guided therapy  Non-PGx guided therapy | 12/13  1/13 |
| Older adults (≥ 65 years ) | PGx-guided therapy  Non-PGx guided therapy | 5/6  1/6 |
| Initiation of patient referral, n (%) | Medical specialist  General practitioner  Pharmacist | 17 (59%)  4 (14%)  8 (27%) |
| Suspected drug-gene-interaction, n  median [IQR]  maximum, minimum | In Total  Per patient | 157  5 [4-7]  11, 1 |
| Reason for suspected drug-gene-interaction, n (%) | Adverse drug reaction  Therapy failure  Adverse drug reaction + therapy failure | 61 (39%)  68 (43%)  28 (18%) |
| Suspected drugs classified according to ATC 3^rd^ level, n (%) | N06A: Antidepressants  N02B: Non-opioid analgesics  M01A: Anti-inflammatory and antirheumatic products, non-steroids  N02A: Opioids  A02B: Drugs for peptic ulcers and gastroesophageal reflux disease  Others^a^ | 36 (23%)  36 (23%)  24 (15%)  14 (9%)  10 (6%)  37 (24%) |
| Confirmed drug-gene-interaction, n (%)  median [IQR]  maximum, minimum | In Total  Per patient | 60 (38%)  2 [1-3]  5, 0 |
| Confirmed drugs classified according to ATC 3^rd^ level, n (%) | N06A: Antidepressants  N02B: Non-opioid analgesics  M01A: Anti-inflammatory and antirheumatic products, non-steroids  N02A: Opioids  A02B: Drugs for peptic ulcers and gastroesophageal reflux disease  Others^b^ | 19 (31%)  4 (7%)  6 (11%)  11 (18%)  8 (13%)  12 (20%) |
| Confirmed involved genes, n (%) | CY2C19  CYP2D6  CYP2C9  Others^c^ | 15 (25%)  12 (20%)  7 (12%)  26 (43%) |
| PGx-based medication change, n (%)  median [IQR]  maximum, minimum | In Total  Per patient | 49 in 23 patients (79%)  2 [1-2]  5, 0 |
| Type of medication changes, n (%) | Initiation of a new drug  Discontinuation of a drug  Dosage adaptation  Start/Stop of a new drug^d^ | 27 (55%)  15 (31%)  4 (8%)  3 (6%) |

^a^Others include general anesthetics (N01A), local anesthetics (N01B), antimigraine preparations (N02C), antiepileptics (N03A), antipsychotics (N05A), anxiolytics (N05B), hypnotics and sedatives (N05C), psychostimulants (N06B), psycholeptics and psychoanaleptics in combination (N06C), centrally acting muscle relaxants (M03B). ^b^Others include general anesthetics (N01A), antiepileptics (N03A), antimigraine preparations (N02C), antipsychotics (N05A), psycholeptics and psychoanaleptics in combination (N06C). ^c^Others include *CYP2B6, ABCB1, COMT, OPRM1, NAT-2*. ^d^Initiation of a new drug, which was subsequently discontinued before the follow-up.
