# Supplementary material for: Association of Pharmacogenotyping and Patient-Reported Outcomes in Chronic Pain Management
Source: Health Serv Insights. 2025 Jul 12;18:11786329251356560. doi: 10.1177/11786329251356560 (PMC12255864; doi:10.1177/11786329251356560)
Supplement: sj-docx-6-his-10.1177_11786329251356560 – Supplemental material for Association of Pharmacogenotyping and Patient-Reported Outcomes in Chronic Pain Management [file sj-docx-6-his-10.1177_11786329251356560.docx]

**Supplementary Table 3. Overview of all patients’ EQ-5D-5L values pre-PGx and post-PGx.**

| **DIMENSION** | **pre-PGx** | | **post-PGx** | |
| --- | --- | --- | --- | --- |
|  | n | % | n | % |
| **MOBILITY** | | | | |
| No problems = 1 | 4 | 13,79 | 6 | 20,69 |
| Slight problems = 2 | 6 | 20,69 | 12 | 41,38 |
| Moderate problems = 3 | 8 | 27,59 | 7 | 24,14 |
| Severe problems = 4 | 10 | 34,48 | 3 | 10,34 |
| unable = 5 | 1 | 3,45 | 1 | 3,45 |
| total | 29 | 100,00 | 29 | 100,00 |
| **SELF-CARE** | | | | |
| No problems = 1 | 12 | 41,38 | 18 | 62,07 |
| Slight problems = 2 | 3 | 10,34 | 6 | 20,69 |
| Moderate problems = 3 | 8 | 27,59 | 3 | 10,34 |
| Severe problems = 4 | 4 | 13,79 | 2 | 6,90 |
| unable = 5 | 2 | 6,90 | 0 | 0,00 |
| total | 29 | 100,00 | 29 | 100,00 |
| **USUAL ACTIVITIES** | | | | |
| No problems = 1 | 3 | 10,34 | 8 | 27,59 |
| Slight problems = 2 | 6 | 20,69 | 11 | 37,93 |
| Moderate problems = 3 | 8 | 27,59 | 5 | 17,24 |
| Severe problems = 4 | 8 | 27,59 | 5 | 17,24 |
| unable = 5 | 4 | 13,79 | 0 | 0,00 |
| total | 29 | 100,00 | 29 | 100,00 |
| **PAIN/DISCOMFORT** | | | | |
| No problems = 1 | 0 | 0,00 | 3 | 10,34 |
| Slight problems = 2 | 4 | 13,79 | 10 | 34,48 |
| Moderate problems = 3 | 10 | 34,48 | 11 | 37,93 |
| Severe problems = 4 | 8 | 27,59 | 4 | 13,79 |
| unable = 5 | 7 | 24,14 | 1 | 3,45 |
| total | 29 | 100,00 | 29 | 100,00 |
| **ANXIETY/DEPRESSION** | | | | |
| No problems = 1 | 6 | 20,69 | 14 | 48,28 |
| Slight problems = 2 | 9 | 31,03 | 5 | 17,24 |
| Moderate problems = 3 | 7 | 24,14 | 7 | 24,14 |
| Severe problems = 4 | 5 | 17,24 | 3 | 10,34 |
| unable = 5 | 2 | 6,90 | 0 | 0,00 |
| total | 29 | 100,00 | 29 | 100,00 |

Abbreviations: n, number; PGx, pharmacogenetics.
